# Low baseline plasma PCSK9 level is associated with good clinical outcomes of immune checkpoint inhibitors in advanced non‐small cell lung cancer

**DOI:** 10.1111/1759-7714.14259

**Published:** 2021-12-27

**Authors:** Mengqing Xie, Xin Yu, Xiangling Chu, Huikang Xie, Juan Zhou, Jing Zhao, Chunxia Su

**Affiliations:** ^1^ Department of Oncology Shanghai Pulmonary Hospital & Thoracic Cancer Institute, Tongji University School of Medicine Shanghai China; ^2^ Department of Pathology Shanghai Pulmonary Hospital & Thoracic Cancer Institute, Tongji University School of Medicine Shanghai China

**Keywords:** biomarker, immune checkpoint inhibitors (ICIs), non‐small cell lung cancer (NSCLC), proprotein convertase subtilisin/kexin type 9 (PCSK9)

## Abstract

**Background:**

Proprotein convertase subtilisin/kexin type 9 (PCSK9) is a crucial protein involved in the metabolism of low‐density lipoprotein cholesterol. However, the role of plasma PCSK9 in predicting the efficacy of ICIs in advanced non‐small cell lung cancer (NSCLC) remains to be clarified.

**Methods:**

We retrospectively reviewed the medical records of NSCLC patients who presented at Shanghai Pulmonary Hospital between April 2019 and June 2020. ELISA was conducted to detect the concentration of PCSK9. Clinical efficacy was evaluated according to Response Evaluation Criteria in Solid Tumors (RECIST, version 1.1).

**Results:**

A total of 55 patients were enrolled in the study. The median progression‐free survival (PFS) following treatment with ICIs in all patients was 9.9 months. The optimal threshold of baseline plasma PCSK9 was 232.2 ng/ml. Patients with low baseline plasma PCSK9 had a longer PFS (NR vs. 7.37 months, *p* = 0.017, HR = 0.207, 95% CI: 0.086–0.498) and a better response (ORR 71.4% vs. 43.9%, *p* = 0.075, DCR 100% vs. 80.5%, *p* = 0.098) to ICIs. Younger patients (≤66 years) with a lower PCSK9 had a significantly longer PFS and higher treatment response than those with a high baseline level of PCSK9 (NR vs. 5.83 months, *p* = 0.021, HR = 0.134, 95% CI: 0.044–0.409; ORR 66.7% vs. 30.0%, *p* = 0.106, DCR 100% vs. 75%, *p* = 0.153). The situation was similar in patients who received first‐line therapy (NR vs. 8.97 months, *p* = 0.022, HR = 0.138, 95% CI: 0.047–0.400; ORR 63.6% vs. 46.4%, *p* = 0.480, DCR 100% vs. 89.3%, *p* = 0.545). Multivariate analysis showed that low PCSK9 concentration was independently associated with PFS (*p* = 0.032, HR = 0.201).

**Conclusions:**

Low baseline plasma PCSK9 level may predict good outcomes in patients with advanced NSCLC treated with ICIs.

## Graphical abstract

The association between PCSK9 and ICIs therapy efficacy in NSCLC remains unclear. The optimal cutoff value of baseline plasma PCSK9 was 232.2 ng/ml. Low baseline plasma PCSK9 was associated with longer PFS and better response in ICIs therapy.

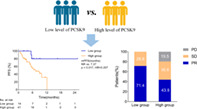

## INTRODUCTION

Immune checkpoint inhibitors (ICIs), particularly inhibitors of the programmed cell death‐1 (PD‐1) axis, have expanded the choice of standard therapeutic options for advanced non‐small cell lung cancer (NSCLC). Pembrolizumab monotherapy has been reported to improve 5‐year overall survival (OS) to 23.2% and 15.5% for treatment‐naive patients and previously treated patients separately.^1^ In addition, the pembrolizumab‐combination group reached a median progression‐free survival (PFS) of 8.8 months, almost two‐fold of that in the placebo‐combination group.^2^ Despite the remarkable success, only a limited number of patients experience a long‐term benefit while the rest develop primary or acquired resistance with a dismal prognosis, creating an urgent need to determine biomarkers for predicting the efficacy and prognosis of patients treated with ICIs.

Many studies have previously explored the predictive or prognostic biomarkers of ICIs in NSCLC. Programmed cell death‐ligand 1 (PD‐L1) expression is so far the only FDA‐approved biomarker for ICI therapy. However, the predictive value of PD‐L1 is unsatisfactory. Even in a population of PD‐L1 tumor proportion score ≥50% patients, the estimated 1‐year survival rate was reported to be 63.4% for combination therapy.^3^ Tumor mutation burden (TMB) is another valuable biomarker in NSCLC. However, the predictive role of TMB needs to be further confirmed due to the inconsistent conclusions regarding its clinical utility from different clinical trials.^4^, ^5^ Other biomarkers such as tumor immune microenvironment (TIME) classification,^6^ tumor‐infiltrating lymphocytes (TILs),^7^ gene expression profiles (GEPs),^8^ and peripheral blood biomarkers^9^ are attracting more attention due to their potential predictive abilities. Given the complexity and multifactorial nature of the antitumor immune response and the mechanisms behind it, the exploration of biomarkers for NSCLC in ICI therapy is becoming crucial at every step, particularly in initial treatment decision‐making.

Proprotein convertase subtilisin/kexin type 9 (PCSK9) has been shown to be a promising molecule in the field of cardiovascular health for several years. It belongs to the proprotein convertases (PCs) family, which can stimulate an inactive secretory precursor into active products.^10^ Mainly secreted from liver cells, PCSK9, also known as neural apoptosis‐regulated convertase 1 (NARC‐1), was first identified in 2003.^11^ Later, the function of PCSK9 in regulating low‐density lipoprotein cholesterol (LDL‐C) metabolism by displaying its vital role in the degradation of LDL receptor was reported.^12^ In addition to the vital role in the cardiovascular area, PCSK9 is also involved in various other physiological processes. For example, studies have expanded the understanding of the role of PCSK9 in cell proliferation and apoptosis. In neuroglioma U251 cell, knockdown PCSK9 plays a vital role in apoptosis, which is confirmed by the activation of caspase‐3 and downregulation of XIAP and p‐Akt.^13^ A similar result was reported in NSCLC cell line A549.^14^ However, in the study by Kai et al., PCSK9 revealed its proapoptotic function in neuronal apoptosis through the modulation of ApoER2.^15^ Due to the apoptosis function of PCSK9 mentioned above, the association between PCSK9 and tumor has recently received greater attention. Liu et al. found that the knockdown of PCSK9 could significantly delay the growth of tumors in the same strain of mice, and enhance the therapeutic effect of ICIs.^16^ Another team found a similar phenomenon but a different mechanism in MC38 and B16 tumors, which also confirms the role of PCSK9 in tumors.^17^ However, the studies which focus on the association between peripheral PCSK9 level and NSCLC are rare. In the present study, we aimed to evaluate the role of baseline plasma PCSK9 level in predicting the efficacy of ICIs in advanced NSCLC patients.

## METHODS

### Study population

We retrospectively collected the electronic medical records of advanced NSCLC patients treated at Shanghai Pulmonary Hospital between April 2019 and June 2020. In total, 55 patients were included in the study. The inclusion criteria were as follows: (1) pathologically‐ or cytologically‐confirmed NSCLC by pathologist, (2) advanced stage IIIb/IV according to the eighth edition of the TNM classification for lung cancer, (3) had received ICI therapy for more than two cycles regardless of treatment lines, (4) Eastern Cooperative Oncology Group performance status (ECOG PS) 0–2, 5 at least one measurable lesion according to Response Evaluation Criteria in Solid Tumors (RECIST, version 1.1) and 6) sufficient organ function. Exclusion criteria included: (1) patients with *EGFR* mutation or ALK/ROS1 rearrangements, (2) incomplete follow‐up information and (3) patients who were enrolled in clinical trials.

### Study design and data collection

This retrospective study was designed to explore the association between baseline plasma PCSK9 level and efficacy of ICI therapy. The clinical data were collected via electronic medical records. All patients received approved anti‐PD‐1 therapy which included pembrolizumab, camrelizumab, toripalimab, sintilimab, and tislelizumab. Combination therapy refers to immune checkpointpoint inhibitors combined with chemotherapy. Pemetrexed‐cisplatin/carboplatin, nab‐paclitaxel‐carboplatin, gemcitabine‐carboplatin were the main chemotherapy regime. Tumor response was evaluated every two cycles of treatment by two independent oncologists. Radiographic complete response (CR), partial response (PR), stable response (SD), and progression disease (PD) were defined according to Response Evaluation Criteria in Solid Tumors (RECIST, version 1.1). Objective response rate (ORR) was defined as CR plus PR. Disease control rate (DCR) was defined as CR plus PR plus SD. PFS was defined as the interval between the initiation of ICI treatment to confirmed disease progression or death. PD‐L1 IHC 22C3 pharmDx assay were used and the positive for PD‐L1 was defined as the PD‐L1 tumor proportion score (TPS) ≥1%. The follow‐up end date was July 30, 2021. The study was approved by the Ethics Committee of Shanghai Pulmonary Hospital. Informed consent of patients was obtained.

### Sample collection and biochemical assays

Peripheral blood samples were obtained by venipuncture before initiation of treatment within three days. They were centrifuged for 15 min at 1000 rpm at a temperature of 4°C within 30 min of collection to obtain the plasma. The plasma was stored at −20°C before detection. Plasma PCSK9 was measured by colorimetric enzyme‐linked immunosorbent assay (ELISA) following the manufacturer's instructions (R&D Systems). The mean minimum detectable dose was 0.096 ng/ml; and mean intra‐ and interassay coefficients of variation were <7%.

### Statistical analysis

SPSS version 23.0 (SPSS Inc.) and GraphPad Prism version 8.0 were used to perform the statistical analyses. X‐tile 3.6.1 software (Yale University, New Haven, CT, USA) was used to determine the optimal threshold for plasma PCSK9 in patients.^18^ Mann–Whitney U test was performed on continuous variables. Pearson's *X* ^2^ or Fisher's exact test was performed on categorical variables. Correlations were performed using Spearman's rank correlation coefficient. The Kaplan–Meier method was used to estimate PFS and the differences between groups were compared by log‐rank test. A two‐sided *p*‐value <0.05 was considered statistically significant. All confidence intervals (CI) were stated at the 95% confidence level. Cox proportional hazards model was used to conduct univariate and multivariate analyses. *p*‐values <0.1 in the univariate analysis were included in the multivariate analysis.

## RESULTS

### Patient characteristics

**FIGURE 1:**
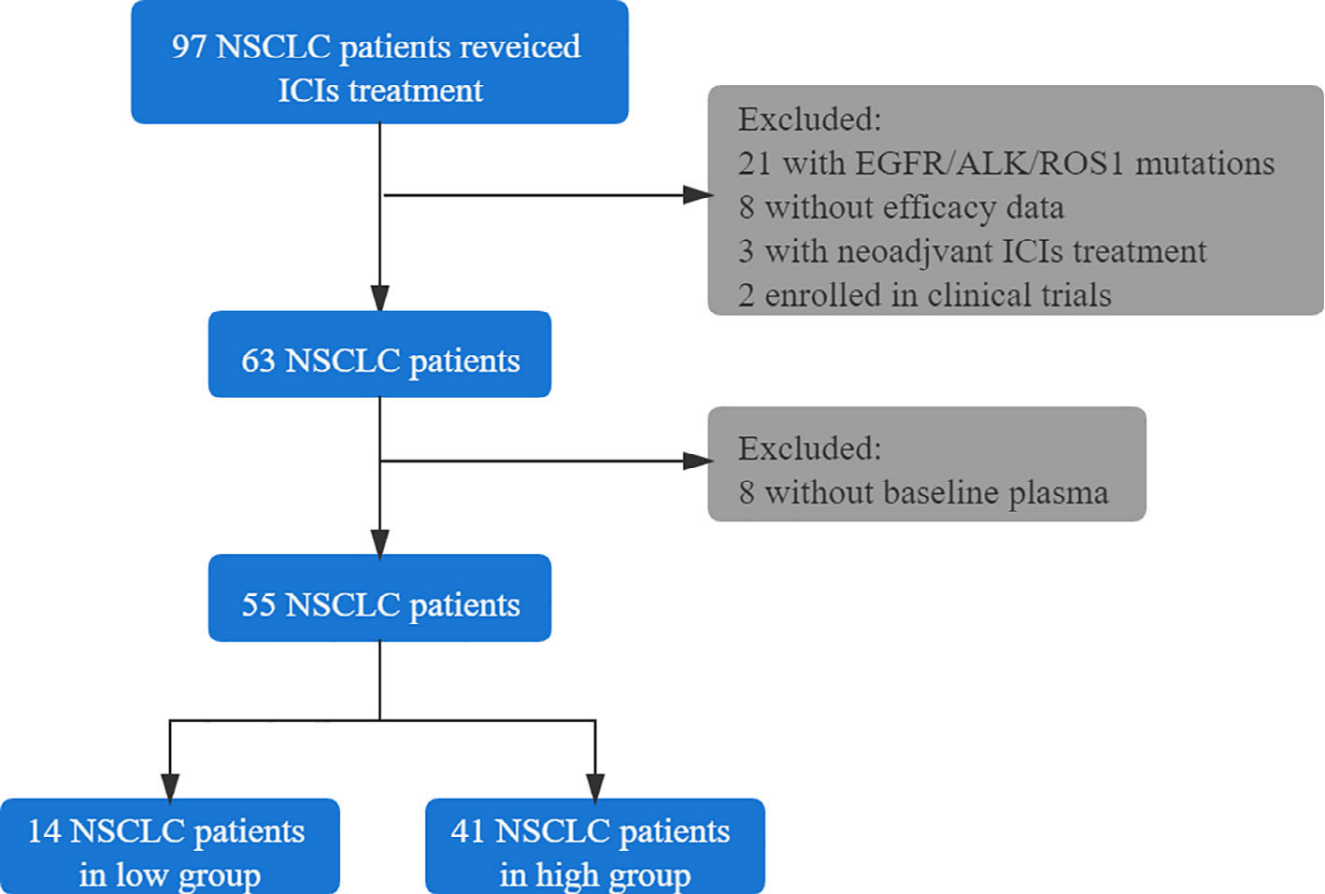
Flow chart of the study. NSCLC, non‐small cell lung cancer; ICI, immune checkpoint inhibitor

A total of 55 patients with advanced NSCLC were enrolled in the study (Figure 1). The median age of all patients was 66 years (range, 48–77 years) and 85.5% were male. The median body mass index (BMI) was 23.1 (range, 16.1–36.1). The majority of patients were stage IV (35, 63.6%) and never smokers (29, 52.7%). A total of 40 patients underwent a test for PD‐L1, 13 (32.5%) were positive and 27 (67.5%) were negative. A total of 15 patients (27.3%) had bone metastasis at baseline. Most patients (39,70.9%) received first‐line ICI combination therapy and all patients (55,100%) had an ECOG PS of 0 or 1. The median follow‐up period was 7.7 months.

**FIGURE 2:**
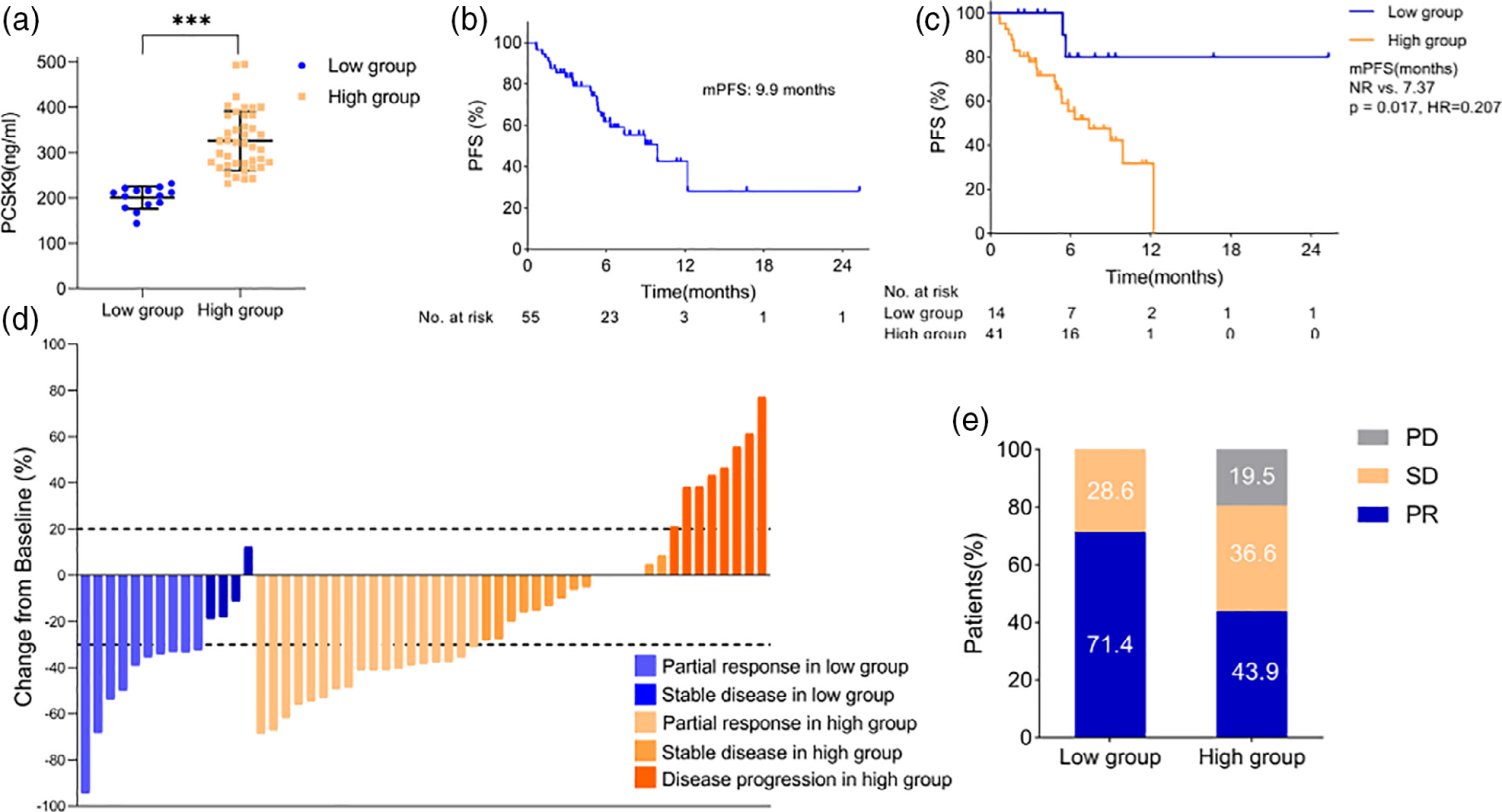
Association between baseline plasma PCSK9 level and efficacy to ICIs therapy. Low group: PCSK9 ≤ 232.2 ng/ml; High group: PCSK9 > 232.2 ng/ml. (a) The baseline plasma PCSK9 level between two groups. (b) PFS of total population enrolled in study. (c) PFS comparison between patients with low level of PCSK9 and high level of PCSK9. (d) Best percent change in the target tumor burden from baseline in total population. (e) Treatment response towards ICI therapy between patients with low and high levels of PCSK9. PCSK9, proprotein convertase subtilisin/kexin type 9; ICIs, immune checkpoint inhibitors; PFS, progress‐free survival

The median baseline plasma PCSK9 level was 279.3 ng/ml (range, 144.3–495.4 ng/ml) in the whole cohort. The optimal threshold level for plasma PCSK9 level was calculated by X‐tile, and the plasma PCSK9 concentration of 232.2 ng/ml provided the best threshold. Thus, 14 patients were categorized to the low PCSK9 group (≤232.2 ng/ml) and 41 patients to the high group (>232.2 ng/ml) (Figure 2a). Among the low group, seven (50.0%) patients were tested for PD‐L1 and four (57.1%) patients were positive. As for the high group, 33 (80.5%) patients had the PD‐L1 test, and nine (27.3%) patients were positive. The baseline characteristics of patients in both groups were well‐balanced. The baseline clinical characteristics of patients are shown in Table 1.

**TABLE 1:** Baseline demographic and clinical characteristics of patients

	All (%) (*n* = 55)	PCSK9 ≤ 232.2 ng/ml (*n* = 14)	PCSK9>232.2 ng/ml (*n* = 41)	*p*‐value
Age (years)				0.316
≤66	29 (52.7)	9 (64.3)	20 (48.8)	
>66	26 (47.3)	5 (35.7)	21 (51.2)	
Gender				0.638
Male	47 (85.5)	13 (92.9)	34 (82.9)	
Female	8 (14.5)	1 (7.1)	7 (17.1)	
BMI				0.279
<25	36 (65.5)	7 (50.0)	29 (70.7)	
≥25	19 (34.5)	7 (50.0)	12 (29.3)	
Smoking history				0.392
Never	29 (52.7)	6 (42.9)	23 (56.1)	
Ever or current	26 (47.3)	8 (57.1)	18 (43.9)	
Histology				0.925
Adenocarcinoma	25 (45.5)	6 (42.9)	19 (46.3)	
Squamous	17 (30.9)	5 (35.7)	12 (29.3)	
NOS	13 (23.6)	3 (21.4)	10 (24.4)	
Stage				0.953
IIIb/IIIc	20 (36.4)	5 (35.7)	15 (36.6)	
IV	35 (63.6)	9 (64.3)	26 (63.4)	
Metastasis site^a^				
Brain	8 (14.5)	1 (7.1)	7 (17.1)	0.638
Bone	15 (27.3)	5 (35.7)	10 (24.4)	0.636
Liver	3 (5.5)	1 (7.1)	2 (4.9)	1.000
No. of treatment lines				0.696
1	39 (70.9)	11 (78.6)	28 (68.3)	
≥ 2	16 (29.1)	3 (21.4)	13 (31.7)	
Treatment regime				1.000
Monotherapy	6 (10.9)	1 (7.1)	5 (12.2)	
Combination therapy	49 (89.1)	13 (92.9)	36 (87.8)	
PD‐L1 status				0.187
Positive	13 (23.6)	4 (28.6)	9 (22.0)	
Negative	27 (49.1)	3 (21.4)	24 (58.5)	
Not clear	15 (27.3)	7 (50.0)	8 (19.5)	
Cholesterol metabolism^b^				
Cholesterol	4.4 (2.9–7.6)	4.8 (3.9–6.3)	4.4 (2.9–7.6)	0.162
Triglyceride	1.4 (0.5–4.3)	1.6 (0.7–2.8)	1.3 (0.5–4.3)	0.388
HDL	1.1 (0.7–2.4)	1.1 (0.8–1.9)	1.1 (0.7–2.4)	0.570
LDL	2.8 (1.4–5.1)	3.2 (2.3–5.1)	2.7 (1.4–4.9)	0.364
Apo A1	1.2 (0.7–2.1)	1.2 (1.0–2.1)	1.3 (0.7–2.0)	0.829
Apo B	1.0 (0.6–1.4)	1.0 (0.6–1.4)	1.0 (0.6–1.3)	0.361
Apo E	38.1 (19.0–80.6)	38.6 (26.2–54.4)	38.5 (19.0–80.6)	0.683
Lp(a)	176.2 (10.4–1083.4)	152.2 (20.2–884.6)	246.5 (10.4–1083.4)	0.173
SdLDL	0.9 (0.3–1.8)	1.1 (0.5–1.3)	0.9 (0.3–1.8)	0.054
Lipase	31.2 (21.5–63.8)	32.3 (23.2–45.9)	31.1 (21.5–63.8)	0.743

^a^ Listed here are the number and percentage of patients with corresponding distant metastatic site.

^b^ Data are expressed as median and range.

### Association between baseline plasma PCSK9 level and clinical outcomes of ICIs

The median PFS of the entire cohort was 9.9 months (Figure 2b). The median PFS was longer in the low PCSK9 group than that in the high group (NR vs.7.37 months, *p* = 0.017, HR = 0.207, 95% CI: 0.086–0.498) (Figure 2c). The low group had a better response to ICI therapy, but the difference was not significant (ORR 71.4% vs. 43.9%, *p* = 0.075, DCR 100% vs. 80.5%, *p* = 0.098) (Figure 2d, e).

**FIGURE 3:**
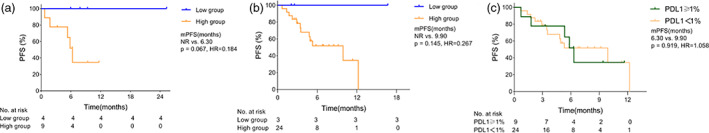
PFS of different PD‐L1 expression. (a) PFS for patients PD‐L1 ≥ 1%. (b) PFS for patients PD‐L1 <1%. (c) PFS for patients with high level of baseline PCSK9. PD‐L1, programmed cell death‐ligand 1

Due to the vital role of PD‐L1 in ICIs therapy, we divided patients into PD‐L1 positive and negative groups. Low baseline PCSK9 level was associated with longer median PFS in both groups, although the difference was not significant (NR vs. 6.30 months, *p* = 0.067, HR = 0.184, 95% CI: 0.030–1.124; NR vs. 9.90 months, *p* = 0.145, HR = 0.267, 95% CI: 0.045–1.578) (Figure 3a, b). In addition, in the high PCSK9 group, patients with PD‐L1 ≥ 1% showed a shorter median PFS compared with those <1%, but with no significant difference (6.30 vs. 9.90 months, *p* = 0.919, HR = 1.058, 95% CI: 0.361–3.096) (Figure 3c).

**FIGURE 4:**
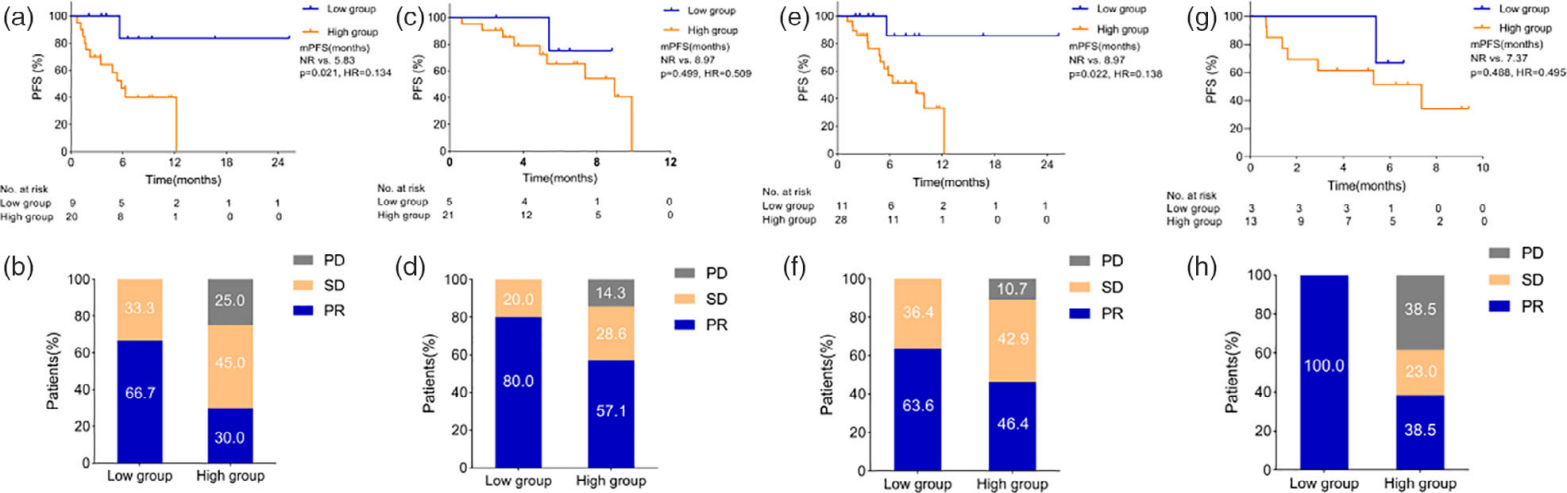
PFS and treatment response of subgroups in total population. Younger patients: ≤66; Elder patients >66. (a) PFS for younger patients. (b) Treatment response for younger patients. (c) PFS for elder patients. (d) Treatment response for elder patients. (e) PFS for patients with first‐line treatment. (f) Treatment response for patients with first‐line treatment. (f) PFS for patients with subsequent treatment. (h) Treatment response for patients with subsequent treatment

Younger patients (≤66 years) with a low level of PCSK9 had a longer median PFS and a higher treatment response than those with a high level of PCSK9 (NR vs. 5.83 months, *p* = 0.021, HR = 0.134, 95% CI: 0.044–0.409; ORR 66.7% vs. 30.0%, *p* = 0.106, DCR 100% vs. 75%, *p* = 0.153) (Figure 4a, b), but the difference was not observed in elder patients (>66 years) (NR vs. 8.97 months, *p* = 0.499, HR = 0.509, 95% CI: 0.101–2.564; ORR 80.0% vs. 57.1*%*, *p* = 0.617, DCR 100% vs. 85.7%, *p* = 1.000) (Figure 4c, d). In addition, for patients receiving first‐line treatment, the low PCSK9 group had a longer PFS and a higher treatment response than the high group (NR vs. 8.97 months, *p* = 0.022, HR = 0.138, 95% CI: 0.047–0.400; ORR 63.6% vs. 46.4%, *p* = 0.480, DCR 100% vs. 89.3%, *p* = 0.545) (Figure 4e, f), while there was no difference in patients who received subsequent lines of ICIs (NR vs. 7.37 months, *p* = 0.488, HR = 0.495, 95% CI: 0.094–2.611; ORR 100% vs. 38.5%, *p* = 0.200, DCR 100% vs. 61.5%, *p* = 0.509) (Figure 4g, h).

In addition, we performed correlation analysis between plasma PCSK9 level and peripheral biochemical indicators due to the role of PCSK9 in cholesterol metabolism reported in previous studies. As shown in Table 2, baseline PCSK9 level was significantly correlated with baseline lipoprotein (a) (Lp[a]) level (*r* = 0.409, *p* = 0.003). Kaplan–Meier analysis was also conducted but no significant differences were found between the two groups with different levels of cholesterol metabolism.

**TABLE 2:** Correlations between baseline PCSK9 level and cholesterol metabolism

PCSK9	*r*	*p*
Cholesterol (mmol/l)	−0.057	0.696
Triglyceride (mmol/l)	−0.189	0.189
HDL (mmol/l)	0.051	0.726
LDL (mmol/l)	0.065	0.655
Apo A1 (g/l)	−0.054	0.708
Apo B (g/l)	0.016	0.911
Apo E (g/l)	0.025	0.865
Lp(a) (mg/l)	0.409	0.003
SdLDL (mmol/l)	−0.217	0.129
Lipase (u/l)	0.003	0.982

### Univariate and multivariate analysis of clinical characteristics for clinical outcomes

The univariate analysis for PFS suggested that combination therapy (*p* = 0.056, HR = 0.344) and low level of PCSK9 (≤232.2 ng/ml) (*p* = 0.03, HR = 0.197) were associated with longer median PFS. The multivariate analysis showed that only PCSK9 concentration was independently associated with PFS (*p* = 0.032, HR = 0.201). The detailed information of univariate and multivariate analysis is shown in Table 3.

**TABLE 3:** Univariate and multivariate analyses of clinical parameters of PFS in patients

Factors	Univariate analyses			Multivariate analyses		
	HR	95% CI	*p*‐value	HR	95% CI	*p*‐value
Age (years)						
≤66/>66	0.995	0.426–2.325	0.991			
Gender						
Male/female	0.978	0.330–2.902	0.968			
BMI						
<25/≥25	1.274	0.498–3.257	0.614			
Smoking history						
Ever or current/never	0.855	0.367–1.990	0.716			
History						
Adeno/NOS	2.133	0.593–7.673	0.246			
Squa/NOS	2.607	0.695–9.780	0.155			
PD‐L1						
Positive/negative	0.667	0.233–1.907	0.449			
Stage						
IIIb or IIIc/IV	0.703	0.288–1.713	0.438			
Metastasis site^a^						
Bone (yes/no)	1.175	0.482–2.865	0.722			
No. of treatment lines						
1/≥2	0.582	0.240–1.410	0.231			
Treatment regime						
Combination/monotherapy	0.344	0.115–1.028	0.056	0.360	0.120–1.076	0.067
PCSK9, ug/ml						
≤232.2/>232.2	0.197	0.045–0.855	0.030	0.201	0.046–0.872	0.032

a Brain and liver metastasis were not included here due to the limited number of patients.

## DISCUSSION

The results of our study suggest that patients with a low baseline level of PCSK9 have a longer PFS and a better response to ICIs. They also showed that the optimal threshold of PCSK9 was 232.2 ng/ml. In subgroup analysis, for patients of a younger age (≤66 years), or those who received first‐line ICI therapy, a low level of PCSK9 was found to be associated with longer PFS. To the best of our knowledge, this is the first study to report that low baseline plasma PCSK9 level is associated with a good outcome to ICI therapy in advanced NSCLC patients.

The low group of PCSK9 in our study displays a better outcome to ICIs therapy than that in the high group, which is consistent with the findings before.^19^ Recent preclinical studies have also suggested that inhibiting PCSK9 can boost the response of tumors to ICIs. Also, Liu et al. carried out experiments in syngeneic mice inoculated with PCSK9‐deficient tumor cells and found that PCSK9 deficiency could synergize with anti‐PD1 antibody.^16^ Further exploration in the tumor microenvironment confirmed the enrichment of CD8^+^ T cells, CD4^+^ T cells and natural killer cells in PCSK9‐deficient tumors. Our study provides a clinical view of the role of PCSK9 in ICI therapy. PD‐L1 is currently the most common biomarker for ICI therapy, and we found that patients with low baseline plasma PCSK9 level had better responses to ICIs regardless of PD‐L1 level. Thus, further studies are needed to explore the combination of PD‐L1 and PCSK9 as biomarkers for ICI therapy in NSCLC. In the subgroup analysis, we found that younger patients (≤66 years) in the low PCSK9 group had a longer PFS than those in the high group, while this phenomenon was not observed in the elder group. Studies have found that ageing interacts with cholesterol metabolism through several mechanisms including lipoprotein dynamics, cholesterol synthesis, etc.^20^, ^21^ Thus, the difference in cholesterol metabolism between groups may be one reason, but this needs further research. In addition, the known function of PCSK9 in cholesterol metabolism triggered us to explore the association between PCSK9 level and cholesterol metabolism in our cohort.^22^ However, only Lp(a), an atherogenic low‐density lipoprotein‐like particle associated with atherosclerotic cardiovascular disease, correlated with baseline PCSK9 levels which are consistent with the findings of previous studies.^23^, ^24^

Many studies have focused on the function of PCSK9 in tumor. Lan et al. analyzed the gene expression and pathways change after treating HepG2 cells with gain‐of‐function PCSK9 at different times.^25^ They found PCSK9 could regulate pathways involving cell cycle and inflammation response in an independent of cholesterol uptake manner. In addition, Piao et al. explored the effects of knockdown and overexpression of PCSK9 on apoptosis of human neuroglioma U251 cells and found that knockdown PCSK9 promoted apoptosis via mitochondrial pathway.^13^ Xu et al. transfected A549, a cell line of human lung adenocarcinoma, with PCSK9 small interfering (si)RNA and found that PCSK9 siRNA could inhibit proliferation and promote apoptosis of A549 cells by inducing endoplasmic reticulum stress and mitochondrial dysfunction.^14^ The experiments in vitro mentioned above provide the potential to explore the effects of PCSK9 in vivo. Momtazi‐Borojeni et al. induced a 4 T1 breast tumor model in mice and injected a nanoliposomal anti‐PCSK9 vaccine according to treatment protocols.^26^ After observing the tumor size, weight, and survival for 60 days, they found that the nanoliposomal anti‐PCSK9 vaccine could alleviate tumor growth by 21.2%, and prolong survival by 4.2%, even though the difference was not significant. Yang et al. found that tumors could block the LDLR‐mediated circulation of the TCR receptor through PCSK9, and weaken the ability of CD8^+^T cells to kill the tumor cells. They focused on the regulatory roles of the PCSK9/LDLR/TCR axis, and found that inhibiting PCSK9 in a genetically or pharmacological way could alleviate the suppressive effect on CD8^+^T cells by preventing the degradation of LDLR. What is more, the combination of PCSK9 inhibitors and ICIs therapy was found to enhance the outcome of malignant tumors, which suggests that there is an underlying association between PCSK9 level and efficacy of ICI therapy.^17^

Collectively, in this study, we enrolled NSCLC patients to explore the role of baseline plasma PCSK9 as a biomarker for ICI therapy. Patients with low baseline level of PCSK9 had a better response to ICI therapy, which has enabled the development of PCSK9‐based biomarkers or clinical drugs. For complete recognition, the application of PCSK9 in the field of NSCLC still has a long way to go. However, this article provides a certain direction. Based on previous studies, the combination of PCSK9 inhibitors and ICIs therapy in NSCLC may be a promising treatment therapy in the future. There are several limitations in our studies. First, this is a single‐center retrospective research with a moderate sample size, and the threshold value of baseline PCSK9 also requires validation in other cohorts. In addition, the overall survival in our cohort was not mature enough, and further follow‐up is needed. Third, the ICIs used in patients were different, which may influence outcomes.

In conclusion, this real‐world retrospective study found that low baseline plasma PCSK9 level was associated with good outcomes of ICIs therapy in advanced NSCLC. PCSK9 could therefore be a promising biomarker for ICI therapy.

## CONFLICT OF INTEREST

The authors report no declarations of interest.
